# Data for developing computerized adaptive testing of problematic mobile phone use

**DOI:** 10.1016/j.dib.2024.110746

**Published:** 2024-07-22

**Authors:** Yaojie Gao, Xiaorui Liu, Zhao Zhou, Miao Chao, Tour Liu

**Affiliations:** a Key Research Base of Humanities and Social Sciences of the Ministry of Education, Academy of Psychology and Behavior, Tianjin Normal University, Tianjin, China; b Faculty of Psychology, Tianjin Normal University, Tianjin, China; c Center of Collaborative Innovation for Assessment and Promotion of Mental Health, Tianjin Normal University, Tianjin, China

**Keywords:** Problematic mobile phone use, Nomophobia, Mobile phone addiction, Scale

## Abstract

With the popularity of smart terminals and mobile Internet, mobile phone had been a necessity in everyone's daily life. Problematic mobile phone use (PMPU) also needs attention. Although there were multiple measurements to assess the PMPU, most of the tools were in traditional forms with fixed items. Therefore, the current study aimed to develop a computerized adaptive testing for problematic mobile phone use (CAT-PMPU) based on the Item Response Theory, which might help to provide an optimal solution to psychological assessments with long-scale and heterogeneous samples. The data in this article provided results of PMPU with different measurements. The dataset provided in this article includes 1619 participants, encompassing middle school students, undergraduate students, and graduate students recruited from various grades in China. The sample consists of 628 males and 991 females. Self-report measures were administrated including the Chinese version of the Nomophobia Questionnaire, Smartphone Addiction Proneness Scale, Smartphone Addiction Inventory, Mobile Phone Addiction Scale, Mobile Phone Addiction Tendency Scale, Smartphone Addiction Scale for College Students, and the Smartphone Addiction Scale for Chinese Adults. All the measurements were in the Chinese version. A .csv file consists of major variables we used was included as supplementary material on the Zenodo Repository. The discussion of the findings based on the dataset could be found in two articles: Development of a Computerized Adaptive Test for Problematic Mobile Phone Use & Generalizing computerized adaptive testing for problematic mobile phone use from Chinese adults to adolescents.

Specifications Table

**Table:** 

Subject	Psychology
Specific subject area	Psychology (General)
Type of data	Microsoft Excel Comma Separated value document(.csv), Table, Figure, Microsoft Word document (.docx)
Data collection	This data was collected using a combination of offline surveys and online surveys [Wen Juan Xing App (https://www.wjx.cn)]. In the online survey, the IP address of the devise was recorded to prevent participants from participating multiple times. Convenience sampling was used. All participants were informed of the study purpose and provided consent to participate.
Data source location	Tianjin Normal University, Tianjin, China
Data accessibility	Respository name: zenodo Data identification number: doi: 10.5281/zenodo.10625336 Direct link to the dataset: https://zenodo.org/records/10625336
Related research article	Lei, G., Xiaorui, L., & Tour, L. (2023). Generalizing computerized adaptive testing for problematic mobile phone use from Chinese adults to adolescents. Current Psychology, 1-11. https://doi.org/10.1007/s12144-023-05447-7

## 1 Value of the Data

• This dataset provides crucial multidimensional information on problematic mobile phone use (PMPU) among middle school, undergraduate, and graduate students in China.
• This dataset can assist researchers in comparing results related to PMPU across different scales, as well as in conducting comparisons and validations of PMPU outcomes among individuals with varying educational backgrounds and age groups.
• These data could be used in structural equation model (SEM), regression analysis, machine learning models, item response model (IRT), and exploration of computerized adaptive testing (CAT), among other analyses.
• Given the global attention to PMPU, this dataset collected within the cultural context of China contributes to cross-cultural and cross-sample research.

## 2 Background

As reported by Digital Reports in 2023, a total of 5.44 billion people use mobile phones, constituting 68 percent of the global population [1]. With the growing ubiquity of smartphones and the internet, problematic mobile phone use (PMPU) has also increased, leading to a series of adverse consequences. The premise for conducting relevant research is the accurate measurement of PMPU. Although there are numerous scales available for measuring PMPU, these tools often rely on Classical Test Theory (CTT), struggling to strike a balance between efficiency and precision. More adaptive measurements are required.

Based on the Item Response Theory, the current study aimed to develop a computerized adaptive testing for problematic mobile phone use (CAT-PMPU). CAT might help to provide an optimal solution to psychological assessments with long-scale and heterogeneous samples. The data in this article provided results of PMPU on seven different measurements.

## 3 Data Description

The .csv file we supplied presents the data on Fear of being unable to obtain information, Fear of losing convenience, Fear of losing contact, Fear of losing the Internet connection, Disturbance of Adaptive Functions, Virtual Life Orientation, Withdrawal, Tolerance, Compulsive behavior, Functional impairment, Withdrawal, Tolerance, Inability to Control Craving, Feeling Anxious & Lost, Withdrawal/Escape, Productivity Loss, withdrawal symptoms, salience, social comfort, mood changes, withdrawal behavior, salience behavior, social comfort, negative effects, Withdrawal, salience, social impairment, somatic discomfort among middle school, undergraduate, and graduate students in China. The data was collected online using Wen Juan Xing App (https://www.wjx.cn) and offline collection. Convenience sampling was used. Specifically, online, we distribute questionnaire links through social media platforms such as WeChat for participants to fill out. Offline, we mainly distribute paper-and-pencil questionnaires at the researcher's school and some collaborating friends' schools for students to answer. Due to the distribution method via Wen Juan Xing App, it is not possible to calculate response rates or reminder frequencies. However, all the data we have obtained are free from missing values, and all items requiring reverse scoring have been appropriately reversed. The Chinese-version questionnaires and their translated version were provided as supplementary files. The further discussions on the research based on this dataset can be referred to in the following two articles: Development of a Computerized Adaptive Test for Problematic Mobile Phone Use & Generalizing computerized adaptive testing for problematic mobile phone use from Chinese adults to adolescents.

Table 1 showed the descriptive results of the grade variable in the dataset.

**Table 1:** Descriptive results of the grade variable in the dataset.

Grade	N	Percent
1 middle school	738	45.58%
2 undergraduate	654	40.40%
3 graduate students	227	14.02%

Table 2 showed the descriptive statistics for Chinese Version of the Nomophobia Questionnaire.

**Table 2:** Descriptive statistic results of Chinese Version of the Nomophobia Questionnaire.

Variables	N	Minimum	Maximum	Mean	SD
1 Nomophobia Scale for Chinese(NMP-C)	1619	16.00	112.00	55.01	22.72
2 Fear of being unable to obtain information	1619	4.00	28.00	12.85	5.93
3 Fear of losing convenience	1619	4.00	28.00	13.30	6.29
4 Fear of losing contact	1619	4.00	28.00	15.84	6.86
5 Fear of losing the Internet connection	1619	4.00	28.00	13.01	6.57

Table 3 showed the descriptive statistics for Smartphone Addiction Proneness Scale and Smart-phone Addiction Inventory.

**Table 3:** Descriptive statistic results of Smartphone Addiction Proneness Scale and Smartphone Addiction Inventory.

Variables	N	Minimum	Maximum	Mean	SD
1 Smartphone Addiction Proneness Scale(SAPS)	1619	10.00	40.00	21.41	4.97
2 Disturbance of Adaptive Functions	1619	1.00	4.00	2.69	0.80
3 Virtual Life Orientation	1619	2.00	8.00	3.65	1.20
4 Withdrawal	1619	4.00	16.00	8.27	2.38
5 Tolerance	1619	3.00	12.00	6.79	2.00
6 Smartphone Addiction Inventory(SPAI)	1619	20.00	80.00	41.77	10.16
7 Compulsive behavior	1619	8.00	32.00	16.21	4.40
8 Functional impairment	1619	7.00	28.00	14.52	4.03
9 Withdrawal	1619	4.00	16.00	8.75	2.18
10 Tolerance	1619	2.00	8.00	4.21	1.36

Table 4 showed the descriptive statistics for Mobile Phone Addiction Scale and Mobile Phone Addiction Tendency Scale.

**Table 4:** Descriptive statistic results of Mobile Phone Addiction Scale and Mobile Phone Addiction Tendency Scale.

Variables	N	Minimum	Maximum	Mean	SD
1 Mobile Phone Addiction Scale(MPAS)	1619	11.00	55.00	24.65	8.68
2 Inability to Control Craving	1619	3.00	15.00	5.34	2.25
3 Feeling Anxious & Lost	1619	4.00	20.00	9.13	3.91
4 Withdrawal/Escape	1619	3.00	15.00	7.52	3.18
5 Productivity Loss	1619	1.00	5.00	2.66	1.18
6 Mobile Phone Addiction Tendency Scale(MPATS)	1619	16.00	80.00	36.99	12.13
7 withdrawal symptoms	1619	6.00	30.00	14.51	4.77
8 salience	1619	4.00	20.00	9.01	3.48
9 social comfort	1619	3.00	15.00	6.59	2.70
10 mood changes	1619	3.00	15.00	6.88	2.67

Table 5 showed the descriptive statistics for Smartphone Addiction Scale for College Students and Smartphone Addiction Scale for Chinese Adults.

**Table 5:** Descriptive statistic results of Smartphone Addiction Scale for College Students and Smartphone Addiction Scale for Chinese Adults.

Variables	N	Minimum	Maximum	Mean	SD
1 Smartphone Addiction Scale for College Students(SAS-C)	1619	11.00	55.00	25.90	8.77
2 withdrawal behavior	1619	2.00	10.00	4.86	1.98
3 salience behavior	1619	3.00	15.00	6.37	2.58
4 social comfort	1619	2.00	10.00	4.32	1.83
5 negative effects	1619	4.00	20.00	10.36	4.12
6 Smartphone Addiction Scale for Chinese Adults(SAS-CA)	1619	14.00	70.00	34.16	11.48
7 Withdrawal	1619	5.00	25.00	12.29	4.70
8 salience	1619	3.00	15.00	6.73	2.63
9 social impairment	1619	3.00	15.00	7.42	3.02
10 somatic discomfort	1619	3.00	15.00	7.73	3.13

Table 6 showed the correlations among seven scales.

**Table 6:** Correlation matrix among seven scales.

	NMP-C	SAPS	SPAI	MPAS	MPATS	SAS-C	SAS-CA
1 Chinese Version of the Nomophobia Questionnaire(NMP-C)	1	.560⁎⁎	.547⁎⁎	.740⁎⁎	.724⁎⁎	.614⁎⁎	.688⁎⁎
2 Smartphone Addiction Proneness Scale(SAPS)		1	.714⁎⁎	.610⁎⁎	.669⁎⁎	.609⁎⁎	.673⁎⁎
3 Smartphone Addiction Inventory(SPAI)			1	.646⁎⁎	.708⁎⁎	.775⁎⁎	.792⁎⁎
4 Mobile Phone Addiction Scale (MPAS)				1	.745⁎⁎	.722⁎⁎	.766⁎⁎
5 Mobile Phone Addiction Tendency Scale(MPATS)					1	.767⁎⁎	.804⁎⁎
6 Smartphone Addiction Scale for College Students(SAS-C)						1	.864⁎⁎
7 Smartphone Addiction Scale for Chinese Adults(SAS-CA)							1

⁎⁎ *p* < .01.

Table 7 showed the correlations among all dimensions from each scale.

**Table 7:** Correlation matrix all dimensions from each scale.

	1	2	3	4	5	6	7	8	9	10	11	12	13	14	15	16	17	18	19	20	21	22	23	24	25	26	27	28
1 Fear of being unable to obtain information	1	.740⁎⁎	.659⁎⁎	.728⁎⁎	.207⁎⁎	.334⁎⁎	.481⁎⁎	.440⁎⁎	.483⁎⁎	.443⁎⁎	.567⁎⁎	.321⁎⁎	.423⁎⁎	.675⁎⁎	.580⁎⁎	.514⁎⁎	.639⁎⁎	.550⁎⁎	.565⁎⁎	.612⁎⁎	.635⁎⁎	.470⁎⁎	.537⁎⁎	.419⁎⁎	.685⁎⁎	.520⁎⁎	.465⁎⁎	.491⁎⁎
2 Fear of losing convenience		1	.677⁎⁎	.753⁎⁎	.203⁎⁎	.380⁎⁎	.534⁎⁎	.447⁎⁎	.485⁎⁎	.431⁎⁎	.577⁎⁎	.299⁎⁎	.421⁎⁎	.674⁎⁎	.551⁎⁎	.471⁎⁎	.622⁎⁎	.545⁎⁎	.618⁎⁎	.624⁎⁎	.633⁎⁎	.434⁎⁎	.522⁎⁎	.382⁎⁎	.705⁎⁎	.515⁎⁎	.437⁎⁎	.485⁎⁎
3 Fear of losing contact			1	.725⁎⁎	.165⁎⁎	.231⁎⁎	.389⁎⁎	.294⁎⁎	.341⁎⁎	.315⁎⁎	.474⁎⁎	.205⁎⁎	.264⁎⁎	.602⁎⁎	.505⁎⁎	.420⁎⁎	.525⁎⁎	.459⁎⁎	.471⁎⁎	.462⁎⁎	.577⁎⁎	.328⁎⁎	.408⁎⁎	.314⁎⁎	.546⁎⁎	.389⁎⁎	.338⁎⁎	.390⁎⁎
4 Fear of losing the Internet connection				1	.193⁎⁎	.360⁎⁎	.533⁎⁎	.387⁎⁎	.480⁎⁎	.422⁎⁎	.572⁎⁎	.306⁎⁎	.402⁎⁎	.676⁎⁎	.566⁎⁎	.424⁎⁎	.650⁎⁎	.569⁎⁎	.598⁎⁎	.601⁎⁎	.659⁎⁎	.486⁎⁎	.555⁎⁎	.356⁎⁎	.690⁎⁎	.522⁎⁎	.400⁎⁎	.451⁎⁎
5 Disturbance of Adaptive Functions					1	.100⁎⁎	.199⁎⁎	.347⁎⁎	.236⁎⁎	.257⁎⁎	.213⁎⁎	.177⁎⁎	.091⁎⁎	.194⁎⁎	.176⁎⁎	.255⁎⁎	.227⁎⁎	.184⁎⁎	.176⁎⁎	.228⁎⁎	.183⁎⁎	.192⁎⁎	.176⁎⁎	.263⁎⁎	.227⁎⁎	.201⁎⁎	.269⁎⁎	.264⁎⁎
6 Virtual Life Orientation						1	.596⁎⁎	.476⁎⁎	.487⁎⁎	.380⁎⁎	.513⁎⁎	.365⁎⁎	.349⁎⁎	.403⁎⁎	.331⁎⁎	.269⁎⁎	.465⁎⁎	.366⁎⁎	.464⁎⁎	.445⁎⁎	.385⁎⁎	.426⁎⁎	.432⁎⁎	.270⁎⁎	.490⁎⁎	.433⁎⁎	.303⁎⁎	.280⁎⁎
7 Withdrawal							1	.556⁎⁎	.564⁎⁎	.436⁎⁎	.618⁎⁎	.405⁎⁎	.360⁎⁎	.556⁎⁎	.438⁎⁎	.347⁎⁎	.595⁎⁎	.458⁎⁎	.563⁎⁎	.553⁎⁎	.544⁎⁎	.451⁎⁎	.482⁎⁎	.294⁎⁎	.644⁎⁎	.467⁎⁎	.350⁎⁎	.358⁎⁎
8 Tolerance								1	.682⁎⁎	.574⁎⁎	.586⁎⁎	.533⁎⁎	.403⁎⁎	.449⁎⁎	.395⁎⁎	.499⁎⁎	.528⁎⁎	.416⁎⁎	.477⁎⁎	.540⁎⁎	.438⁎⁎	.484⁎⁎	.454⁎⁎	.491⁎⁎	.556⁎⁎	.494⁎⁎	.562⁎⁎	.485⁎⁎
9 Compulsive behavior									1	.840⁎⁎	.712⁎⁎	.654⁎⁎	.547⁎⁎	.516⁎⁎	.447⁎⁎	.535⁎⁎	.634⁎⁎	.518⁎⁎	.593⁎⁎	.599⁎⁎	.535⁎⁎	.649⁎⁎	.590⁎⁎	.595⁎⁎	.646⁎⁎	.616⁎⁎	.623⁎⁎	.613⁎⁎
10 Functional impairment										1	.648⁎⁎	.619⁎⁎	.517⁎⁎	.454⁎⁎	.390⁎⁎	.525⁎⁎	.568⁎⁎	.460⁎⁎	.534⁎⁎	.571⁎⁎	.476⁎⁎	.652⁎⁎	.551⁎⁎	.656⁎⁎	.572⁎⁎	.605⁎⁎	.635⁎⁎	.709⁎⁎
11 Withdrawal											1	.531⁎⁎	.449⁎⁎	.594⁎⁎	.483⁎⁎	.448⁎⁎	.711⁎⁎	.583⁎⁎	.622⁎⁎	.608⁎⁎	.635⁎⁎	.536⁎⁎	.584⁎⁎	.464⁎⁎	.689⁎⁎	.562⁎⁎	.498⁎⁎	.515⁎⁎
12 Tolerance												1	.413⁎⁎	.353⁎⁎	.317⁎⁎	.407⁎⁎	.437⁎⁎	.375⁎⁎	.437⁎⁎	.429⁎⁎	.366⁎⁎	.590⁎⁎	.462⁎⁎	.523⁎⁎	.451⁎⁎	.457⁎⁎	.499⁎⁎	.478⁎⁎
13 Inability to Control Craving													1	.556⁎⁎	.443⁎⁎	.433⁎⁎	.476⁎⁎	.428⁎⁎	.482⁎⁎	.469⁎⁎	.414⁎⁎	.546⁎⁎	.507⁎⁎	.467⁎⁎	.496⁎⁎	.520⁎⁎	.509⁎⁎	.464⁎⁎
14 Feeling Anxious & Lost														1	.650⁎⁎	.539⁎⁎	.678⁎⁎	.592⁎⁎	.643⁎⁎	.627⁎⁎	.689⁎⁎	.510⁎⁎	.557⁎⁎	.435⁎⁎	.717⁎⁎	.565⁎⁎	.481⁎⁎	.477⁎⁎
15 Withdrawal/Escape															1	.511⁎⁎	.566⁎⁎	.509⁎⁎	.516⁎⁎	.541⁎⁎	.521⁎⁎	.456⁎⁎	.546⁎⁎	.413⁎⁎	.594⁎⁎	.497⁎⁎	.424⁎⁎	.467⁎⁎
16 Productivity Loss																1	.459⁎⁎	.375⁎⁎	.420⁎⁎	.478⁎⁎	.446⁎⁎	.459⁎⁎	.438⁎⁎	.615⁎⁎	.537⁎⁎	.466⁎⁎	.676⁎⁎	.566⁎⁎
17 withdrawal symptoms																	1	.745⁎⁎	.703⁎⁎	.756⁎⁎	.715⁎⁎	.593⁎⁎	.659⁎⁎	.458⁎⁎	.765⁎⁎	.678⁎⁎	.510⁎⁎	.534⁎⁎
18 salience																		1	.676⁎⁎	.682⁎⁎	.681⁎⁎	.539⁎⁎	.630⁎⁎	.369⁎⁎	.648⁎⁎	.578⁎⁎	.433⁎⁎	.455⁎⁎
19 social comfort																			1	.732⁎⁎	.694⁎⁎	.683⁎⁎	.646⁎⁎	.481⁎⁎	.757⁎⁎	.615⁎⁎	.491⁎⁎	.516⁎⁎
20 mood changes																				1	.660⁎⁎	.669⁎⁎	.636⁎⁎	.526⁎⁎	.748⁎⁎	.685⁎⁎	.547⁎⁎	.554⁎⁎
21 withdrawal behavior																					1	.573⁎⁎	.634⁎⁎	.480⁎⁎	.786⁎⁎	.582⁎⁎	.484⁎⁎	.503⁎⁎
22 salience behavior																						1	.645⁎⁎	.644⁎⁎	.650⁎⁎	.654⁎⁎	.607⁎⁎	.585⁎⁎
23 social comfort																							1	.524⁎⁎	.690⁎⁎	.628⁎⁎	.535⁎⁎	.526⁎⁎
24 negative effects																								1	.541⁎⁎	.547⁎⁎	.738⁎⁎	.636⁎⁎
25 Withdrawal																									1	.678⁎⁎	.581⁎⁎	.606⁎⁎
26 salience																										1	.621⁎⁎	.608⁎⁎
27 social impairment																											1	.674⁎⁎
28 somatic discomfort																												1

Note:

* *p <* .05.

⁎⁎ *p* < .01.

**Fig. 1:**
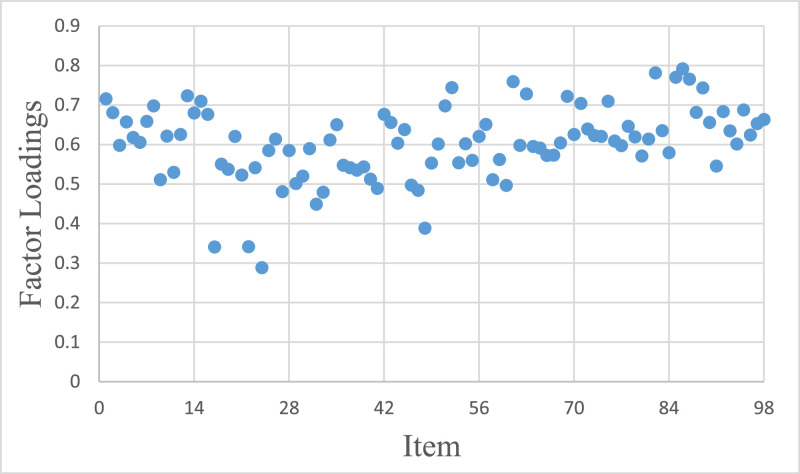
Factor Loadings of 98 Items on the First Principal Component.

Figure 1 presents the factor loadings of 98 items on the first principal component. Subsequent results do not include items with first principal component factor loadings less than 0.4 (including SAPS_1, SAPS_6, SAPS_8, MPAS_2). Exploratory factor analysis (EFA) was conducted on the remaining items, revealing that all item factor loadings were greater than 0.4. The first eigenvalue and the second eigenvalue were 36.606 and 6.213 respectively, with a ratio of 5.89. Additionally, the first factor explained variance was 37.353%.

Table 8 presents the results of fitting the retained items to two commonly used polytomous score models, GRM and GPCM.

**Table 8:** Model fit indices.

Models	AIC	BIC	Loglik
GRM	356612.5	359172.6	-177831.3
GPCM	360700.0	363260.0	-179875.0

**Fig. 2:**
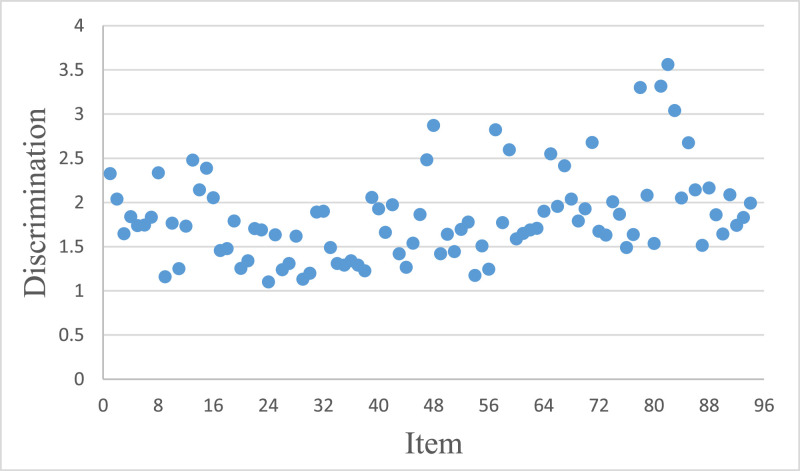
Item Discrimination Estimated by GRM.

Figure 2 illustrates the item discrimination estimated by GRM. Furthermore, the difficulty range of the item bank is [-1.95, 3.97].

**Fig. 3:**
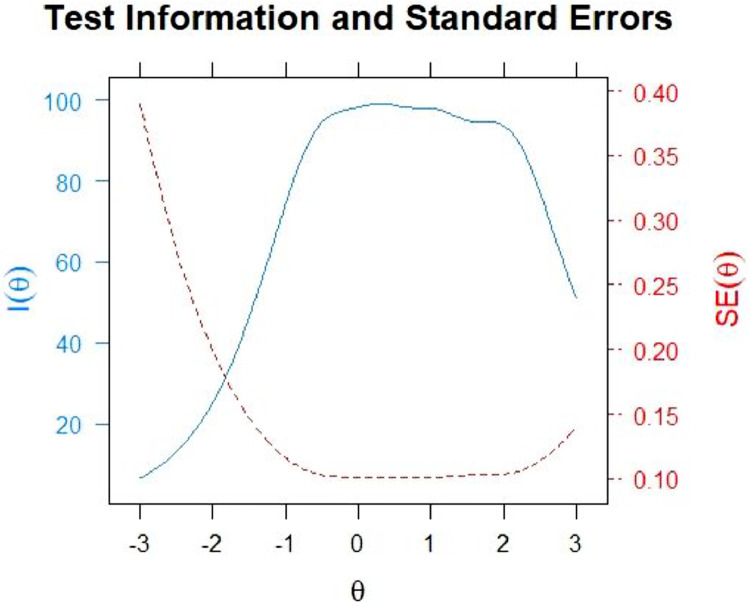
Information and Standard Error of the Item Bank.

Figure 3 illustrates the information and standard error of the item bank.

## 4 Experimental Design, Materials and Methods

### 4.1 Participants

The research data includes a total of 1619 participants from China, comprising both middle school students, undergraduate students, and graduate students. It is worth mentioning that this study does not have specific inclusion or exclusion criteria for participants. Just as we aim to provide a new technical means for screening and assessing problematic mobile phone use in a large population, our participants are not limited to any specific group. However, participants who spend less than 200 seconds answering or provide erroneous responses are excluded. After excluding data with missing values and anomalous responses, the final dataset consists of 1619 participants with complete data (*M*_age_ = 18.80, *SD* = 2.86). Among them, there are 628 males (38.8%) and 991 females (61.2%). For a detailed breakdown of participant grades, please refer to Table 1.

### 4.2 Questionnaires

During the testing process, participants were required to respond to seven scales related to problematic mobile phone use. To minimize the burden on participants and ensure the accuracy of the measurement results, we invited five senior psychology graduate students and two experienced psychology teachers to review and eliminate redundant items. Specifically, items with semantic similarity (e.g., SAPS's disturbance of adaptive functions sub-dimension, 6 items from SPAI, 6 items from MPAS, 11 items from SAS-C) and items unrelated to problematic mobile phone use (e.g., SAS-CA's dimensions for app usage and app updates) were removed. Through their evaluation, we ensured the content validity of the remaining 98 items.

#### 4.2.1 Chinese version of the nomophobia questionnaire (NMP-C)

To measure nomophobia, we used the Nomophobia Questionnaire [2]. The instrument was developed by Yildirim and Correia [3]. The Chinese version was revised by Ren et al. and demonstrated good structural validity (χ^2^/df = 3.91, RMSEA = 0.067, TLI = 0.941, CFI = 0.952, SRMR = 0.04) [2]. The questionnaire consisted 16 items in four dimensions: fear of not being able to communicate, fear of losing Internet connection (especially social media), fear of not being able to access information, and fear of giving up convenience. Each dimension had 4 items. Participants rated each item on a seven-point scale from 1 = strongly disagree to 7 = strongly agree. The Cronbach's alpha coefficient in this study was 0.956.

#### 4.2.2 Smartphone addiction proneness scale (SAPS)

The Smartphone Addiction Proneness Scale was used and demonstrated good structural validity (NFI = 0.943, TLI = 0.902, CFI = 0.902, RMSEA = 0.034) [4]. The scale assessed individuals’ smartphone addiction proneness in four dimensions: disturbance of adaptive functions, virtual life orientation, withdrawal, and tolerance. The subscale contained 10 items. Participants rated each item on a four-point scale (1 = strongly disagree, 4 = strongly agree). The Cronbach's alpha coefficient in the current study was 0.837.

#### 4.2.3 Smartphone Addiction Inventory (SPAI)

The Smartphone Addiction Inventory by Lin et al. [5] was used. The scale contains 20 items in four dimensions, compulsive behavior, functional impairment, withdrawal, and tolerance. Participants rated each item on a five-point scale (1 = Very inconsistent, 5 = Very well suited to). The Chinese version was shown to have good internal consistency and conceptual validity. The Cronbach's alpha coefficients in this study was 0.926.

#### 4.2.4 Mobile phone addiction scale (MPAS)

The Mobile Phone Addiction Scale [6]was used and demonstrated good structural validity. The scale contains 11 items in four dimensions, inability to control craving, feeling anxious and lost, withdrawal and escape, and productivity loss. Participants rated each item on a five-point scale (1 = never, 5 = always). The Cronbach's alpha coefficients in this study was 0.888.

#### 4.2.5 Mobile phone addiction tendency scale (MPATS)

The Mobile Phone Addiction Tendency Scale [7] was used and confirmed to have good structural validity (CFI = 0.960, RMSEA = 0.070, NFI = 0.940, IFI = 0.960, RFI = 0.930). The scale contains 16 items in four dimensions, withdrawal symptoms, salience, social comfort, and mood changes. Participants rated each item on a five-point scale (1 = Very inconsistent, 5 = Very well suited to). The Cronbach's alpha coefficients in this study was 0.923.

#### 4.2.6 Smartphone addiction scale for college students (SAS-C)

The Smartphone Addiction Scale for College Students by Su et al. [8] was used. It had satisfying structure validity (χ^2^/df = 1. 57, CFI = 0. 92, IFI = 0. 93, RMSEA = 0. 05, SRMR < 0. 001). The scale contains 11 items in four dimensions, withdrawal behavior, salience behavior, social comfort, and negative effects. Participants rated each item on a five-point scale (1 = Strongly unacceptable, 5 = Strongly acceptable). The Cronbach's alpha coefficients in this study was 0.905.

#### 4.2.7 Smartphone addiction scale for Chinese adults (SAS-CA)

The Smartphone Addiction Scale for Chinese Adults by Chen et al. [9] was used. It had satisfying structure validity (χ^2^/df = 2.13, CFI = 0.94, IFI = 0.94, RMSEA = 0.043). The scale contains 14 items in four dimensions, withdrawal, salience, social impairment, and somatic discomfort. Participants rated each item on a five-point scale (1 = Strongly unacceptable, 5 = Strongly acceptable). The Cronbach's alpha coefficients in this study was 0.933.

## 5 Statistical Analysis

The results of descriptive statistics (Mean and SD) and correlations among the major variables were presented in Table 2, Table 3, Table 4, Table 5, Table 6, Table 7.

## Limitations

Although there is a predominance of female participants in our data, we still have abundant and sufficient male samples, and currently there is no evidence indicating that gender would have an impact on the quality and utilization of this data.

## Ethics Statement

This study was approved by the ethics committee of Tianjin University (XL2020-12). The questionnaire was anonymous, and all participants were informed of the study purpose and provided consent. For underage participants (under 18 years old), informed consent has been obtained from their parents or legal guardians.

## CRediT authorship contribution statement

**Yaojie Gao:** Formal analysis, Writing – review & editing, Project administration. **Xiaorui Liu:** Formal analysis, Resources, Methodology. **Zhao Zhou:** Methodology. **Miao Chao:** Conceptualization, Writing – original draft. **Tour Liu:** Conceptualization, Supervision, Project administration.

## Data Availability

DATA_Problematic Mobile Phone Use CAT (Original data) (zenodo).

## Appendix Supplementary materials

Image, application 1

## References

[1] Digital Reports (2023). data from: https://datareportal.com/reports/digital-2023-global-overview-report

[2] Ren S., Gu Li., Liu T. (2020). Revisement of nomophobia scale for Chinese. J. Psychol. Explor..

[3] Yildirim C., Correia A. (2015). Exploring the dimensions of nomophobia: development and validation of a self-reported questionnaire. Comput. Hum. Behav..

[4] Kim D., Lee Y., Lee J., Nam J., Chung Y. (2014). Development of Korean smartphone addiction proneness scale for youth. PLoS ONE.

[5] Lin Y., Chang L., Lee Y., Tseng H., Kuo T., Chen S. (2014). Development and validation of the smartphone addiction inventory (SPAI). PLoS ONE.

[6] Leung L. (2008). Linking psychological attributes to addiction and improper use of the mobile phone among adolescents in Hong Kong. J. Child. Media.

[7] Xiong J., Zhou Z., Chen W., You Z., Zhai Z. (2012). Development of the mobile phone addiction tendency scale for college students. Chin. Ment. Health J..

[8] Su S., Pan T., Liu Q., Chen X., Wang Y., Li M. (2014). Development of the smartphone addiction scale for college students. Chin. Ment. Health J..

[9] Chen H., Wang L., Qiao N., Cao Y., Zhang Y. (2017). Development of the smartphone addiction scale for Chinese adults. Chin. J. Clin. Psychol..

