# Supplementary material for: Data for developing computerized adaptive testing of problematic mobile phone use
Source: Data Brief. 2024 Jul 22;55:110746. doi: 10.1016/j.dib.2024.110746 (PMC11342194; doi:10.1016/j.dib.2024.110746)
Supplement: Supplementary file 1 [file mmc1.docx]

CAT for Assessing Problematic Mobile Phone Use Item Bank

**Basic Information**

**Gender**:**1 =**Male, **2** =Female

**Grade**: **1 =** Freshmen, **2 =** Sophomore, **3 =** Junior, **4 =** Senior, **5 =** Grade 1 master, **6 =** Grade 2 master, **7 =** Grade 3 master, **8 =** doctor, 11 = Grade 7 students, 12 = Grade 8 students, 13 = Grade 9 students, 14 = Grade 10 students, 15 = Grade 11 students, 16 = Grade 12 students

**Age**

# Chinese Version of the Nomophobia Questionnaire（NMP-C）

7-point Likert-type (from 1 = Not meet at all to 7 = Completely in conformity with)

1. I feel uncomfortable if I don't have continuous access to information through my mobile phone.

2. I would be annoyed if I could not look information up on my mobile phone when I wanted to do.

3. Not being able to get news (e.g., happenings, weather, etc.) on my mobile phone makes me nervous.

4. I get annoyed when I can't use my mobile phone and/or its capabilities when I want to.

5. I get scared when my mobile phone battery runs out.

6. I will feel panic if my phone reaches its monthly data limit.

7. If the phone has no signal or can't connect to Wi-Fi, I will keep checking it.

8. If I can't use my mobile phone, I will be afraid of getting into trouble.

9. If my mobile phone is not around, I will worry that my family and friends will not be able to contact me.

10. I feel nervous when my mobile phone is not around and I cannot receive messages and calls.

11. When my mobile phone is not around and I can't keep in touch with my family and friends, I will be worried.

12. If I did not have my mobile phone with me, I would be nervous because I could not know if someone had tried to get a hold of me.

13. When my mobile phone is not around and I am disconnected from the network, I will be nervous.

14. If I did not have my mobile phone with me, I would be uncomfortable because I could not stay up-to-date with social media and online networks.

15. If I did not have my mobile phone with me, I would feel awkward because I could not check the network connection and online network update notification.

16. When my mobile phone is not around, I will feel anxious because I can't check my QQ, WeChat, email and other messages.

- Fear of being unable to obtain information: 1, 2, 3, 4
- Fear of losing convenience: 5, 6, 7, 8
- Fear of losing contact: 9, 10, 11, 12
- Fear of losing the Internet connection: 13, 14, 15, 16

# 中文版无手机恐惧量表（NMP-C）

采用李克特7点计分（1=完全不符合，7=完全符合）

1.如果不能通过我的手机持续访问信息，我会感到不舒服

2.当我想要在我手机上查看信息却无法查看时，我会很生气

3.不能在手机上获得新闻（例如，发生的事情，天气等）会让我感到紧张

4.当我想要使用手机和/或它的功能却不能使用时，我就会很生气

5.手机电量用完会让我感到害怕

6.如果手机达到了每月的数据流量限制，我会感到恐慌

7.如果手机没有信号或者无法连接到无线网络，我会不停地检查

8.如果我不能使用我的手机，我会害怕陷入困境

9.当手机不在身边导致家人和朋友无法联系到我时，我会很担心

10.当手机不在身边导致不能接收短信和电话时，我会感到紧张

11.当手机不在身边导致无法与家人朋友保持联系时，我会很着急

12.当手机不在身边导致我不知道是否有人想要联系我时，我会感到很紧张

13.当手机不在身边导致我与网络脱离时，我会很紧张

14.当手机不在身边导致我无法赶得上社交媒体和在线网络的发展时，我会感到不舒服

15.当手机不在身边导致我不能检查网络连接和在线网络更新的通知时，我就会觉得难受

16.当手机不在身边导致我无法检查我的 QQ、微信、电子邮件等的信息时，我会感到焦虑

- 害怕无法获得信息：1, 2, 3, 4
- 害怕失去便利：5, 6, 7, 8
- 害怕失去联系：9, 10, 11, 12
- 害怕失去网络连接：13, 14, 15, 16

# Smartphone Addiction Proneness Scale（SAPS）

4-point Likert-type (from 1 = Strongly disagree to 4 = Strongly agree)

1. Spending time on mobile phones makes me happier than spending time with family and friends.

2. I feel very painful if I am not allowed to play mobile phone.

3. I feel lost the whole world when I can't use the mobile phone.

4. I get restless without mobile phone.

5. I can't control the time of using mobile phone.

6. Even without a mobile phone, I won't worry.

7. Even when I think I should stop using my mobile phone, I will continue to use it and cannot stop.

8. Mobile phones won't distract me when studying.

9. I feel very panic when I can't use my mobile phone.

10. It has become a habit of me to spend a lot of time on my mobile phone.

- Disturbance of Adaptive Functions: 8
- Virtual Life Orientation: 1, 3
- Withdrawal: 2, 4, 6, 9
- Tolerance: 5, 7, 10

# 智能手机成瘾倾向量表（SAPS）

采用李克特4点计分（1=非常不同意，4=非常同意）

1将时间花在玩手机上，比与家人和朋友待在一起会更让我开心

2如果不被允许玩手机的话，我会感到非常痛苦

3当我不能使用手机时，我会觉得失去了整个世界

4当没有手机时，我会变得焦躁不安

5我不能控制使用手机的时间

6即使没有手机，我也不会焦虑

7即使当我认为应该停止使用手机时，我仍会继续使用，无法停止

8在学习时，手机不会分散我的注意力

9当我不能使用手机时，我会感到很恐慌

10将大量时间花在手机上已经成了我的一种习惯

- 适应功能障碍：8
- 虚拟生活取向：1, 3
- 戒断症状：2, 4, 6, 9
- 耐受性：5, 7, 10

# Smartphone Addiction Inventory（SPAI）

4-point Likert-type (from 1 = Strongly disagree to 4 = Strongly agree)

1. Someone has told me more than once that I spend too much time on my phone.

2. I feel uncomfortable after I stop using my mobile phone for a period of time.

3. As long as I play mobile phone immediately will be energetic no matter how tired before.

4. The amount of time I spent on my mobile phone and the extra cost caused by it were more than I expected.

5. Although mobile phone use has a negative impact on my interpersonal relationship, the time spent online has not decreased.

6. Sleep time is less than 4 hours due to playing with mobile phones. This has happened to me more than once.

7 In the last three months, the time I use my mobile phone has obviously increased

8. I will feel depressed and disappointed after stop using mobile phone for a period of time.

9. I can't control my impulse to play mobile phone.

10. I found that I had less time to get along with and communicate with my friends due to my addiction to mobile phones.

11. I feel eye discomfort and back pain due to excessive mobile phone use .

12. Mobile phone use has a certain negative impact on my study and work performance.

13. I feel that I have missed some messages after stop using my mobile phone for a period of time.

14. My interaction with my family is reduced because of playing mobile phone.

15. My entertainment activities have been reduced due to playing mobile phone.

16. I will have the impulse to open it again after I stop using mobile phone.

17. I tried to spend less time on mobile phone, but efforts were in vain.

18. Playing with mobile phones has become a habit. My sleep time and sleep quality have decreased.

19. I need to spend a lot of time on mobile phone to achieve the same satisfaction as before.

20. If I didn't have mobile phone, I couldn't have meal.

- Compulsive behavior: 3, 4, 5, 8, 9, 10, 15, 17
- Functional impairment: 6, 10, 11, 12, 14, 18, 19
- Withdrawal: 2, 13, 16, 20
- Tolerance: 1, 7

# 智能手机成瘾量表（SPAI）

采用李克特4点计分（1=非常不同意，4=非常同意）

1有人不止一次的告诉我，我花在手机上的时间太多了

2当停止使用手机一段时间后，我会感到不舒服

3无论之前有多疲劳，只要一玩手机我立马就会精力充沛

4我使用手机的时长和由于手机而产生的额外花费都比我预想的要多

5尽管使用手机给我的人际关系带来了负面影响，但是上网时间没有减少

6由于玩手机而导致睡眠时间不足4小时，此事在我身上发生了不止一次

7最近3个月内，我使用手机的时间明显变长了

8当我停止使用手机一段时间以后，我会感到沮丧和失望

9我无法控制玩手机的冲动

10我发现由于自己沉迷手机而减少了与朋友们的相处和交流

11由于过度使用手机，我感到眼睛不适和背部酸痛

12使用手机对我的学业和工作表现产生了一定的负面影响

13当停止使用手机一段时间以后，我会感觉错过了一些消息

14由于玩手机我和家人的互动减少了

15由于玩手机，我的娱乐活动减少了

16在停止使用手机后我会有再次想打开它的冲动

17我曾尝试过在手机上花费更少的时间，但最终的努力都是徒劳的

18玩手机已经成了一个习惯，我的睡眠时间减少了，睡眠质量也下降了

19我需要在手机上花费大量的时间才能达到与以前一样的满意度

20如果没有手机的话，我会吃不下饭

- 强迫行为：3, 4, 5, 8, 9, 10, 15, 17
- 功能障碍：6, 10, 11, 12, 14, 18, 19
- 戒断症状：2, 13, 16, 20
- 耐受性：1, 7

# Mobile Phone Addiction Scale（MPAS）

5-point Likert-type (from 1 = Never to 5 = Always)

1. I tried to hide from others how long I used my mobile phone

2. I received a mobile phone bill that I couldn't afford

3. I always feel that I don't have enough time to play mobile phone.

4. I find it difficult for me to turn off mobile phone.

5. If I haven't checked the information or turned on mobile phone for a period of time, I will feel very anxious.

6. If I didn't have mobile phone, I would feel very lost.

7. If there is no mobile phone, my friends will find it difficult to get in touch with me.

8. When I feel isolated, I use mobile phone to communicate with others.

9. When I feel lonely, I use mobile phone to communicate with others.

10. When I feel depressed, playing mobile phone will make me better.

11. I found myself on my mobile phone when I should be doing something else, and that did cause some problems.

- Inability to Control Craving: 1, 2, 3
- Feeling Anxious & Lost: 4, 5, 6, 7
- Withdrawal/Escape: 8, 9, 10
- Productivity Loss: 11

# 移动手机成瘾量表（MPAS）

采用李克特5点计分（1= 一点也不，5=总是）

1我曾试图向他人隐瞒自己使用手机的时长

2我收到了自己无法负担得起的手机账单

3我总会觉得玩手机的时间不够

4我发现将手机关机对我来说是一件困难的事情

5如果有一段时间没有查看信息或打开手机，我就会感到非常焦虑

6如果没有手机我会感到非常失落

7如果没有手机，我的朋友会发现很难与我取得联系

8当我觉得自己被孤立时，我会用手机跟其他人交流

9当我觉得自己孤单时，我会用手机和其他人交流

10当我觉得沮丧时，玩手机会让我变好

11当我应该做其他事情时，却发现自己都在玩手机，且这确实造成了一定问题

- 无法控制渴望：1, 2, 3
- 感到焦虑和失落：4, 5, 6, 7
- 戒断症状：8, 9, 10
- 工作效率下降：11

# Mobile Phone Addiction Tendency Scale（MPATS）

5-point Likert-type (from 1 = Very inconsistent to 5 = Very well suited to)

1. If I haven't looked at my mobile phone for a while, I will immediately check whether there is a text message/missed call

2. I would rather choose to chat on my mobile phone than face-to-face communication.

3. When waiting for someone, I always call frequently to ask where the other person is. If I don't call, I will be impatient

4. If I don’t use my phone for a long time, I feel uncomfortable.

5. I can't pay attention in class because of calls and text messages.

6. I would feel lonely without my mobile phone.

7. I feel more confident when communicating with others on my mobile phone.

8. When my phone doesn't ring for a while, I feel uncomfortable and subconsciously check my phone for missed calls/messages.

9. I often have hallucinations of "my cell phone is ringing/my cell phone is vibrating".

10. I feel more fulfilled when I have more phone calls and text messages.

11. I am often afraid of my mobile phone turning off automatically.

12. My mobile phone is a part of me, and when it goes away, I feel like I've lost something.

13. My classmates and friends often say that I rely too much on my phone.

14. When the mobile phone is often disconnected and cannot receive the signal, I get anxious and become irritable.

15. In class, I often take the initiative to focus on the mobile phone and affect the class.

16. I find it more comfortable to communicate with others on my mobile phone.

- Withdrawal symptoms: 1, 4, 6, 8, 10, 12
- Salience: 5, 9, 13, 15
- Social comfort: 2, 7, 16
- Mood change: 3, 11, 14

# 手机成瘾倾向量表（MPATS）

采用李克特5点计分（1= 非常不符合，5=非常符合）

1一段时间没有看手机我会马上去查阅是否有短信/未接来电

2我宁愿选择手机聊天，不愿直接面对面交流

3在等人的时候我总是频繁打电话问对方身在何处，如果不打就焦急难耐

4如果很长时间没用手机，我会觉得难受

5课堂上我会因为电话和短信而不能专心听讲

6如果没有手机我会感到孤独

7用手机与他人交流时，我感到更自信

8一段时间手机铃声不响，我会感到不适应，并下意识看一眼手机是否有未接电话/短信

9我经常有“我的手机铃声响了/我的手机在震动”的幻觉

10电话多，短信多我会觉得生活更充实

11我经常害怕手机自动关机

12手机是我的一部分，一旦减少就觉得失去了什么似的

13同学朋友常说我太过于依赖手机

14当手机经常连不上线，收不到信号时我会焦虑并且脾气变得暴躁起来

15课堂上，我会经常主动把注意力集中在手机上而影响听课

16我觉得用手机跟他人交流更舒适

- 戒断症状：1, 4, 6, 8, 10, 12
- 突显行为：5, 9, 13, 15
- 社交抚慰：2, 7, 16
- 心境改变：3, 11, 14

# Smartphone Addiction Scale for College Students（SAS-C）

5-point Likert-type (from 1 = Strongly unacceptable to 5 = Strongly acceptable)

1. My classmates and friends said that I spent too much time on mobile phone.

2. I feel that I need to spend more time on mobile phone to be satisfied.

3. A direct result of spending time on mobile phone is that my learning efficiency decreases.

4. Friends and family complain that I use mobile phone too much.

5. When I am sad, the first thing I think of is playing mobile phone.

6. If my mobile phone is not around for a period of time, I often worry about missing the calls.

7. Playing mobile phone has affected my academic performance.

8. The delay caused by playing mobile phone has brought me a lot of trouble.

9. If I can't use mobile phone for a period of time, I will feel very anxious.

10. I find it hard to sleep because I use mobile phone to check on my friends.

11. My academic performance has decreased because of playing with mobile phone.

- withdrawal behavior: 6, 9
- salience behavior: 1, 2, 4
- social comfort: 5, 10
- negative effects: 3, 7, 8, 11

# 大学生智能手机成瘾量表（SAS-C）

采用李克特 5 点计分，（1=非常不符合，5=非常符合）

1同学、朋友说我花了太多时间在我的手机上

2我感到我需要花更多时间在我的手机上才能满足

3把时间花在手机上产生的一个直接结果就是我的学习效率下降

4朋友和家人抱怨我过多的使用手机

5当我伤心难过时，最先想到的是玩手机

6如果一段时间我的手机不在身边，我常担心错过了电话

7玩手机影响了我的学习成绩

8因为玩手机而造成的拖延给我带来了很多麻烦

9如果一段时间不能使用手机，我会感到很焦虑

10我会因为用智能手机关心朋友的在线情况而难以入睡

11因为玩手机，我的学习成绩下降了

- 戒断症状：6, 9
- 突显行为：1, 2, 4
- 社交抚慰：5, 10
- 消极影响：3, 7, 8, 11

# Smartphone Addiction Scale for Chinese Adults（SAS-CA）

5-point Likert-type (from 1 = Strongly unacceptable to 5 = Strongly acceptable)

1. Mobile phone is such a big part of my life that I find it hard to sit still without it.

2. If I don't use mobile phone for a period of time, I will feel uneasy.

3. I feel restless and irritable when mobile phone have no signal or Internet access.

4. I want to play mobile phone as soon as I wake up every morning.

5. My life would become very boring without mobile phone.

6. I prefer to chat with my friends and watch web pages on mobile phone, rather than communicate directly with my family or friends in real life.

7. Using mobile phone has a negative impact on my personal safety, such as looking at mobile phone when crossing the road, driving or waiting, resulting in danger.

8. Even when I'm out with my family or eating, I can't stop playing mobile phone.

9. I often fail to complete my work or learning tasks on time because of playing mobile phone.

10. I will delay what I have planned because of mobile phone use.

11. When I am working or studying, it is difficult to concentrate due to mobile phone use.

12. I feel neck pain due to excessive mobile phone use.

13. I have experienced dizziness or eye irritation due to excessive mobile phone use.

14. I don't get enough sleep and feel tired from using mobile phone too much.

- Withdrawal: 1, 2, 3, 4, 5
- Salience: 6, 7, 8
- social impairment: 9, 10, 11
- somatic discomfort: 12, 13, 14

# 成年人智能手机成瘾量表（SAS-CA）

采用李克特 5 点计分，（1=非常不符合，5=非常符合）

1手机是我生活中的重要部分，如果它不在身边，我就会觉得坐立难安

2如果一段时间不使用手机，我会感到不安

3当手机没信号或不能上网的时候，我会感到不安和烦躁

4每天早上一醒来我就会想要玩手机

5如果没有手机，我的生活就会变得很乏味

6我更喜欢在手机上跟我的朋友聊天、看网页、而不是跟现实生活中的家人或者朋友直接交流

7使用手机对我的个人安全产生了负面影响，比如过马路的时候看手机、在开车或者等待的时候看手机，从而导致危险

8即使我和家人参加户外活动或吃饭，我依然控制不住一直玩手机

9我常常因为玩手机而导致工作或者学习任务无法按时完成

10我会由于使用手机而耽误已经计划好的事情

11当我在进行工作或学习任务的时候，由于使用手机而很难集中注意力

12由于过度使用手机，我感到脖子疼

13我由于过度使用手机而出现过头晕或眼睛不舒适的症状

14我由于过度使用手机而缺乏足够的睡眠并感觉到疲惫

- 戒断症状：1, 2, 3, 4, 5
- 凸显行为：6, 7, 8
- 社会社交功能受损：9, 10, 11
- 生理不适：12, 13, 14
